# Prevalence and correlates of diastolic dysfunction in patients with hypertension: a cross-sectional study from in The Kingdom of Saudi Arabia

**DOI:** 10.11604/pamj.2021.40.159.31089

**Published:** 2021-11-16

**Authors:** Sameer Al-Ghamdi, Faisal Khalid Alzubaidi, Sultan Abdulrahman Alharthai, Meshal Saleh Alzahim, Fahad Mohammed Al Bahily, Mohammed Ibrahim Alsifaee, Hiaallah Ali Alshehri, Muath Salman Anazi

**Affiliations:** 1 Department of Family Medicine, College of Medicine, Prince Sattam Bin Abdulaziz University, Al-Kharj 11942, Saudi Arabia,; 2 Shaqra University, Shaqra College of Medicine, Shaqra City, Saudi Arabia,; 3 Imam Muhammad Ibn Saud Islamic University, College of Medicine, Riyadh, Saudi Arabia

**Keywords:** Diastolic dysfunction, hypertension, Saudi Arabia

## Abstract

**Introduction:**

diastolic dysfunction refers to impaired ventricular relaxation or filling regardless of ejection fraction and symptoms. It accounts for 8% and 25% in the hospitalized and general population, respectively. The present study was conducted to determine the prevalence and correlates of diastolic dysfunction in hypertensive patients living in Saudi Arabia.

**Methods:**

a multicentric, cross-sectional study was conducted from February 2019 to February 2020 at King Khalid Hospital and Prince Sultan Center for Health Services, Prince Sattam Bin Abdulaziz University hospital in Al Kharj, and Al Kharj Military Industries Corporation hospital, KSA. All patients with hypertension who underwent an echocardiography were included in the study. Logistic regression analysis was performed to determine factors associated with left ventricular diastolic dysfunction (LVDD).

**Results:**

the study included a total of 104 participants, where 51.9% were females and the mean age of the patients was 48.01±12.81 years. Most patients had an abnormal echocardiography finding (64.4%, n = 67). The most common abnormalities were left ventricular (LV) hypertrophy (44.2%, n = 46), and diastolic dysfunction, (35.6%, n = 37). The study revealed that age (aOR: 6.1, 95% CI 1.17-31.3; p = 0.032) and dyslipidemia (aOR: 3.45, 95% CI 1.16-10.24; p = 0.026) have significant association with LVDD in the patients with hypertension.

**Conclusion:**

in conclusion, diastolic dysfunction is prevalent among older hypertensive patients and those with dyslipidaemia. Age and dyslipidaemia were non-modifiable and modifiable factors associated with LVDD in hypertensive patients, respectively.

## Introduction

Heart failure (HF) is a global pandemic and serious health concern affecting 64.34 million people all over the world, posing significant morbidity, mortality, and expenditure [1]. Approximately 1% of the world´s population is suffering from HF [2]. A 2020 study in Saudi Arabia, estimated the 1-year mortality rate for those with acute and chronic heart failure to be 19.5% and 9%, respectively [2]. The prevalence of HF is rising, and diastolic HF is a major cause of hospital admissions, especially among the elderly women [3]. Diastolic dysfunction refers to impaired ventricular relaxation or filling regardless of ejection fraction and symptoms [4]. Some of the major risk factors for diastolic dysfunction are increasing age, diabetes mellitus, ischemic heart disease, hypertension and left ventricular (LV) hypertrophy [5,6]. Diastolic dysfunction is more prevalent among hypertensive patients [7]. Approximately, 34% of patients with diabetes suffer from diastolic dysfunction [6]. Determinants of diastolic dysfunction include myocardial relaxation, myocardial stiffness, chamber geometry, wall thickness, preload and afterload, passive properties of ventricular wall and heart rate [4].

Diastolic HF causes lesser annual deaths as compared to that of systolic HF [8]. However, diastolic dysfunction is important, and it should be recognized as early as possible to avoid its progression to diastolic failure. Diastolic dysfunction has been reported to be more prevalent in the patients with heart failure with preserved ejection fraction (HFpEF) as compared to those with heart failure with reduced ejection fraction (HFrEF) [9]. The reason is that the patients with HFpEF have extensive perivascular fibrosis as compared to those with HFrEF. The management of diastolic dysfunction is not clearly codified. However, drugs like calcium channel blockers (CCBs), beta blockers (BBs), angiotensin-converting enzyme inhibitors (ACEIs) and angiotensin-receptor blockers (ARBs) play beneficial role in the management of the patients with diastolic dysfunction. Similarly, risk factors for CVDs such as high blood pressure and consequential left ventricular hypertrophy are highly prevalent in Saudi population [10]. The literature on the risk factors of diastolic dysfunction from Saudi Arabia is lacking. Therefore, this study was planned to determine the prevalence and correlates of left ventricular diastolic dysfunction (LVDD) among patients with hypertension attending 3 large tertiary care hospitals in Saudi Arabia.

## Methods

**Study design and setting**: this was a multicentric, cross-sectional study conducted from February 2019 to February 2020 at the cardiology units of three hospitals in the Kingdom of Saudi Arabia (KSA)including King Khalid Hospital and Prince Sultan Center for Health Services, Prince Sattam Bin Abdulaziz University hospital in Al Kharj, KSA, and Al Kharj Military Industries Corporation hospital. These units have adequate human resources and are fully equipped to diagnose and manage the full spectrum of cardiovascular diseases according to international standards.

**Study population**: all patients with previously diagnosed primary hypertension who underwent an echocardiography at the study sites during the study period were evaluated for inclusion into the study. Hypertensive patients showing signs and symptoms of heart failure, or chest x ray findings of structural heart disease or cardiomegaly were indicated to undergo echocardiography at the 3 hospitals. We included males and females of all ages and nationalities. Patients with newly diagnosed hypertension, those with secondary hypertension, those who refused to consent to participate in the study, and those with any data missing from their electronic medical records were excluded. The calculated minimum required sample size was 97 patients. This sample size was calculated using an open Epi sample size calculator, taking the prevalence of LV diastolic dysfunction to be 50.3% among hypertensive patients who underwent echocardiography [11] at margin of error 10% and confidence interval (CI) 95%. Non-probability consecutive sampling technique was used for sampling.

**Data collection**: patient medical information pertaining to their age, gender, nationality, history of present illness, including hypertensive symptoms (such as headache, blurring of vision, nose bleeds, or shortness of breath) and/or complications (such as myocardial infarction, aneurysms, left ventricular hypertrophy, or heart failure), co-morbidities (such as diabetes mellitus, ischemic heart disease, atrial fibrillation), smoking history, blood pressure readings, body mass index (BMI), biochemical tests for dyslipidaemia, and echocardiography findings were collected through a survey of their electronic medical records. The outcome of interest was the presence of echocardiography findings suggestive of LVDD. After at least 5 min of rest, blood pressure (BP) was measured in a sitting position from non-dominant arm placed at the level of the heart, using adults´ cuffs (32-42 cm) adapted to an automated sphygmomanometer. Hypertension was diagnosed based on blood pressure recordings measured according to the 2017 American College of Cardiology (ACC) and American Heart Association (AHA) clinical practice guidelines [12]. Hypertension was classified into normal, elevated, or hypertension stages 1 or 2 according to the ACC and AHA 2017 hypertension cut-offs for systolic blood pressure (SBP) and diastolic blood pressure (DBP) [12]. Normal BP was defined as SBP < 120 mm Hg and DBP < 80 mm Hg; elevated BP as SBP 120-129 mm Hg and DBP <80 mm Hg; hypertension stage 1 as SBP 130-139 or DBP 80-89 mm Hg; and hypertension stage 2 as SBP ≥ 140 mm Hg or DBP ≥ 90 mm Hg[12]. Those patients with a BMI (calculated as weight is kilograms divided by height in metres squared) of 30 and above were categorised as obese, as defined by the WHO [13].

**Statistical analysis**: data was entered and analysed using the Statistical Package for Social Services version 22. Descriptive statistics were obtained for study variables. Mean and standard deviation were computed for quantitative variables i.e., age and BMI. Frequency and percentages were used to summarise qualitative variables. The association of study variables with LVDD was assessed by constructing a binary logistic regression model. Variables with a p value of less than 0.20 in the univariable analysis were included in the multivariable model. Two-tailed p-values <0.05 were considered statistically significant.

**Ethical considerations**: the study was approved by the Medical Ethics Committee of the Prince Sattam Bin Abdulaziz University (Ethics approval number REC-HSD-80-2021) and was conducted according to its guidelines. A written informed consent was obtained from all study participants (or their legal guardians, where applicable) before enrolment into the study. All patient information obtained from electronic health records was completely anonymized.

## Results

**General characteristics**: after exclusion of 25 participants, the study included a total of 104 participants. The study population included mostly non-Saudi nationals, around 60%, where 51.9% were females and the mean age of the patients was 48.01±12.81 years. Most, nearly 80%, were obese or suffered from mild hypertension (HTN). thirty-seven point five percent (37.5%) of participants suffered from ischemic heart disease, 29.8% from dyslipidaemia, while 26% of them had diabetes mellitus (Table 1).

**Table 1:** characteristics of the study population

Study variables			Frequency (%)
**Age group (in years)**	Mean ±Standard deviation		48.01±12.8
	Less than 40		28(26.9%)
	40-59		55(52.9%)
	60 or more		21(20.2%)
**Gender**	Female		54(51.9%)
	Male		50(48.1%)
**Nationality**	Non-Saudi		61(58.7%)
	Saudi		43(41.3%)
**Personal history Obesity**	Smoker		4(3%)
	Yes		82(78.8%)
	No		22(21.2%)
**Fasting Lipid**	Abnormal-Dyslipidaemia present		31 (29.8%)
**Profile**	Normal		73(70.2%)
**Severity of**	Mild		83(79.8%)
**hypertension**	Moderate to Severe		21 (20.2%)
**Co-morbidities**	Diabetes Mellitus		39(37.5%)
	Ischemic heart disease		27(26%)
	Atrial fibrillation		2(1.9%)
**Total**			**104(100%)**

**Echocardiography findings**: Most patients had an abnormal echocardiography finding (64.4%, n = 67). The most common abnormalities were left ventricular (LV) hypertrophy (44.2%), and LVDD, (35.6%) (Table 2).

**Table 2:** echocardiography findings and patterns

Echocardiography findings	Frequency (%)
Abnormal	**67 (64.4%)**
Normal	37 (35.6%)
Left ventricular hypertrophy	**46 (44.2%)**
Diastolic dysfunction	37(35.6%)
Reduced ejection fraction	6 (5.8%)
Tricuspid regurgitation	2(1.9%)
Systolic dysfunction	2 (1.9%)
Mitral regurgitation	1 (1%)
Aortic regurgitation	1 (1%)
Right ventricular hypertrophy	1 (1%)
Total	**104 (100%)**

**Factors associated with diastolic dysfunction**: in the initial univariable analysis age, gender, obesity, severity of hypertension, diabetes mellitus, ischaemia heart disease, dyslipidaemia, and left ventricular hypertrophy reached a statistical significance of p < 0.2. In the multivariable analysis only 2 of the included variables reached statistical significance (Table 3). Participants aged 60 and above, were 6 times more likely to have LVDD than those less than 40 years, (aOR: 6.1, CI: 1.17-31.30, p = 0.032). Those with dyslipidaemia were nearly 3.5 times more likely to have LVDD (aOR: 3.45, CI: 1.16-10.24, p = 0.026).

**Table 3:** correlates of left ventricular diastolic dysfunction

Associated factors		LV diastolic dysfunction		Logistic Regression			
		No	Yes	Unadjusted OR (95%CI)	p value	Adjusted OR (95%CI)	p value
**Age groups**	Less than 40	25 (89.3%)	3 (10.7%)	1		1	
	41-59	33 (60%)	22 (40%)	5.56 (1.19-20.7)	0.011	2.9(0.71-12.6)	0.135
	60 or more	9 (42.9%)	12 (57.1%)	11.1 (2.54-48.7)	0.001	6.1 (1.17-31.3)	0.032
**Gender**	Female	38 (70.4%)	16 (29.6%)	1		1	
	Male	29 (58%)	21 (42%)	1.72 (0.77-3.87)	0.19	1.93(0.69-5.43)	0.213
**Obesity**	No	17 (77.3%)	5 (22.7%)	1		1	
	Yes	50 (61%)	32 (39%)	2.18 (0.73-6.48)	0.163	2.89(0.67-12.45)	0.154
**Nationality**	Non-Saudi	39 (63.9%)	22(36.1%)	1		----	----
	Saudi	28 (65.1%)	15 (34.9%)	0.95 (0.42-2.15)	0.901	----	----
**Severity of hypertension**	Mild	58 (69.9%)	25 (30.1%)	1		1	
	Moderate to severe	9 (42.9%)	12 (57.1%)	3.1 (1.16-8.27)	0.024	0.68 (0.11-4.3)	0.667
**Diabetes Mellitus**	No	48 (73.8%)	17 (26.2%)	1		1	
	Yes	19 (48.7%)	20 (51.3%)	2.97 (1.29-6.9)	0.011	2.59(0.83-8.1)	0.103
**Smoker**	No	65(65%)	35(35%)	1		----	----
	Yes	2 (50%)	2 (50%)	1.9 (0.25-13.8)	0.545	----	----
**Ischemic heart disease**	No	55 (71.4%)	22(28.6%)	1		1	
	Yes	12 (44.4%)	15(55.6%)	3.13 (1.26-7.7)	0.014	1.60 (0.27-9.4)	0.601
**Dyslipidemia**	No	54 (74%)	19 (26%)	1		1	
	Yes	13 (41.9%)	18 (58.1%)	3.94 (1.63-9.5)	0.002	3.45 (1.16-10.24)	0.026
**Left ventricular systolic dysfunction**	No	66 (64.7%)	36 (35.3%)	1		----	----
	Yes	1 (50%)	1 (50%)	1.83 (0.11-30.2)	0.672	----	----
**Tricuspid regurgitation**	No	66 (64.7%)	36(35.3%)	1		----	----
	Yes	1(50%)	1(50%)	1.83 (0.11-30.2)	0.672	----	----
**Aortic regurgitation**	No	66 (64.1%)	37 (35.9%)	1		----	----
	Yes	1(100%)	0 (0%)	0(n/a)	1	----	----
**Mitral regurgitation**	No	66 (64.1%)	37(35.9%)	1		----	----
	Yes	1 (100%)	0 (0%)	0 (n/a)	1	----	----
**Atrial Fibrillation**	No	65(63.7%)	37(36.3%)	1		----	----
	Yes	2(100%)	0 (0%)	0(n/a)	1	----	----
**Heart failure**	No	63 (64.3%)	35 (35.7%)	1		----	----
	Yes	4 (66.7%)	2 (33.3%)	0.9 (0.16-5.16)	0.906	----	----
**Left ventricular hypertrophy**	No	42 (72.4%)	16(27.6%)	1		1	
	Yes	25 (54.3%)	21 (45.7%)	2.21 (0.97-4.9)	0.058	1.21 (0.45-3.23)	0.705

For Multivariate analysis: Adjusted OR (95% CI), Significance level <0.05. Model: Age group, Gender, Obesity, Hypertension, Diabetes mellitus, Ischemic heart disease, dyslipidemia, Left ventricular hypertrophy.

## Discussion

This cross-sectional study was conducted to determine the prevalence and correlates of diastolic dysfunction in hypertensive patients living in Saudi Arabia. The study revealed that age and dyslipidemia have significant association with LVDD in the patients with hypertension. The prevalence of LVDD among hypertensive patients increases with advancing age and more likely to occur in those with dyslipidemia as compared to those without it. It has been observed that LVDD is one of the first noticeable manifestations among the patients with hypertension which can be detected on echocardiography as left ventricular filling abnormality even when it is clinically unnoticeable [14]. A retrospective cohort study including 300 patients with heart failure in a tertiary care center in Saudi Arabia found 84.4% to have left ventricular dysfunction and 33.3% to have hypertensive heart disease [15]. The present study has reported the prevalence of LVDD as 44.2% which increases from mild to severe hypertension. A cross-sectional study has reported LVDD in 82.86% of patients with hypertension. They also reported that that the prevalence of LVDD increases with the grade of hypertension i.e., prevalence of diastolic dysfunction was more among severely hypertensive patients as compared to that among mildly hypertensive patients [7]. Hence, the prevalence of LVDD among Nigerian is much more than those living in Saudi Arabia.

Several correlates of LVDD among hypertensive patients have been reported. A large study has reported that factors associated with LVDD include advanced age, duration of hypertension, tobacco use, high systolic blood pressure, uncontrolled high blood pressure, and slow heart rate [16].Similarly, Swierblewaska *et al*. [12] retrospectively analyzed 610 hypertensive patients of CARE NORTH study (conducted in Poland) to determine the prevalence and severity of LVDD among hypertensive patients. They reported normal diastolic function only in 49.7% patients, demonstrating female gender, advancing age, obesity, diabetes, left ventricular mass index as independent risk factors of LVDD. The present study revealed no significant association of male gender, obesity, severity of hypertension, diabetes mellitus, ischemic heart disease, and LVH with LVDD. Similarly, Zheng *et al*. [17] conducted a cross-sectional study to evaluate metabolic risk factors of LVDD among middle-aged Chinese population. They reported diabetes, hypertension, and abdominal obesity as significant risk factors of LVDD. In this context, situation is different among hypertensive patients living in Saudi Arabia in terms of prevalence of LVDD. Here, we found only age and dyslipidemia as significant correlates of LVDD among hypertensive patients. Moreover, prevalence of LVDD among hypertensive patients in Saudi Arabia lower than that in other countries. However, further studies at large scale can be conducted in other regions of the country to verify the results of the present study.

The strength of the study is that it is the first study, to the best of our knowledge, to perform echocardiographic evaluations for LVDD among hypertensive patients attending tertiary care centers in KSA. It revealed significant associations between age and dyslipidemia with LVDD. This may provoke further studies and certain strategies to adopt to prevent LVDD. The limitation includes small size of the study as large population suffers from hypertension. Further, given that only we only included patients who underwent echocardiographic study for hypertension evaluation, inclusion of patients with more severe hypertensive disease as opposed to those whose hypertension did not warrant echocardiographic evaluation is an inherent sampling bias in this study. This along with a high margin of error of 10% precludes the extrapolation of our research findings to the general Saudi population [18]. Further, due to the very low prevalence of atrial fibrillation in this study population this covariate could not be included in the logistic regression model. This along with non-availability of information regarding sedentary or active lifestyles, alcohol history, co-morbidities other than diabetes/ischemic heart disease, N-terminal pro-B-type natriuretic peptide measurements, and other theoretically relevant factors limit the robustness of the logistic regression model constructed.

## Conclusion

Left ventricular diastolic dysfunction is frequent among hypertensive patients where main risk factors are old age and dyslipidaemia. Elderly participants and those with dyslipidemia were more likely to be diagnosed with LVDD. Therefore, preventive measures and lifestyle modifications would help improve dyslipidaemia among hypertensive patients, reducing the risk of LVDD.

### What is known about this topic

- The prevalence of left ventricular diastolic dysfunction has been reported to be up to 82.86% of patients with hypertension;
- Determinants of diastolic dysfunction include myocardial relaxation, myocardial stiffness, chamber geometry, wall thickness, preload and afterload, passive properties of ventricular wall and heart rate.

### What this study adds

- It is the first study to perform echocardiographic evaluations for LVDD among hypertensive patients attending tertiary care centers in KSA.
- The prevalence of left ventricular diastolic dysfunction as 44.2%;
- Age and dyslipidemia have significant association with left ventricular diastolic dysfunction among the patients with hypertension in Saudi Arabia.
